# Risk Factors for Postpartum Hemorrhage in a Thai–Myanmar Border Community Hospital: A Nested Case-Control Study

**DOI:** 10.3390/ijerph18094633

**Published:** 2021-04-27

**Authors:** Waraporn Thepampan, Nuchsara Eungapithum, Krittai Tanasombatkul, Phichayut Phinyo

**Affiliations:** 1 Labor Unit, Maesai Hospital, Chiang Rai 57130, Thailand; Dang-wara@hotmail.com; 2 Research and Development Division, Maesai Hospital, Chiang Rai 57130, Thailand; eungyong58@gmail.com; 3 Department of Family Medicine, Faculty of Medicine, Chiang Mai University, Chiang Mai 50200, Thailand; krittaikt@gmail.com; 4 Center for Clinical Epidemiology and Clinical Statistics, Faculty of Medicine, Chiang Mai University, Chiang Mai 50200, Thailand; 5 Musculoskeletal Science and Translational Research (MSTR) Cluster, Chiang Mai University, Chiang Mai 50200, Thailand

**Keywords:** risk factors, pregnancy, postpartum hemorrhage, etiology, developing countries

## Abstract

Postpartum hemorrhage (PPH) is a common complication of pregnancy and a global public health concern. Even though PPH risk factors were extensively studied and reported in literature, almost all studies were conducted in non-Asian countries or tertiary care centers. Our study aimed to explore relevant risk factors for PPH among pregnant women who underwent transvaginal delivery at a Thai–Myanmar border community hospital in Northern Thailand. An exploratory nested case-control study was conducted to explore risk factors for PPH. Women who delivered transvaginal births at Maesai hospital from 2014 to 2018 were included. Two PPH definitions were used, which were ≥ 500 mL and 1000 mL of estimated blood loss within 24 h after delivery. Multivariable conditional logistic regression was used to identify significant risk factors for PPH and severe PPH. Of 4774 women with vaginal births, there were 265 (5.55%) PPH cases. Eight factors were identified as independent predictors for PPH and severe PPH: elderly pregnancy, minority groups, nulliparous, previous PPH history, BMI ≥ 35 kg/m^2^, requiring manual removal of placenta, labor augmentation, and fetal weight > 4000 gm. Apart from clinical factors, particular attention should be given to pregnant women who were minority groups as PPH risk significantly increased in this population.

## 1. Introduction

Postpartum hemorrhage (PPH) is a common complication of pregnancy and a major cause of maternal mortality worldwide [1]. The ACOG reVITALize program defined PPH as a total blood loss of more than or equal to 1000 mL, or any blood loss accompanied by clinical signs and symptoms of hypovolemic shock within 24 h after the birth process. However, in practice, a blood loss of more than 500 mL should be considered precautious, and early management should be initiated [2,3]. The overall prevalence of PPH in South-Eastern Asia was previously reported at 4.88% [4]. In Thailand, the figures varied from 1.4% to 10.6%, depending on the types of health care settings [5]. The highest prevalence was usually identified in district hospitals where healthcare resources were limited, and the distribution of risk factors was different from hospitals in the urban area [5,6].

PPH is a life-threatening, yet preventable, condition [7]. Several studies have reported risk factors that are associated with PPH, which were generally classified according to the labor period: antepartum factors (e.g., advanced maternal age, multiple pregnancies, history of previous PPH, taking an anticoagulant drug, and maternal anemia [8,9]), and intrapartum factors (e.g., labor induction and augmentation, prolonged third stage of labor, lacerations of birth passage, and fetal macrosomia [10,11]). Although some patients might develop PPH without any risk factor [12], determining the maternal risk factors for PPH is still important [13]. Pregnant women with multiple risks for PPH should be closely monitored and given early intervention to prevent the occurrence of PPH (e.g., active management of the third stage of labor (AMTSL) with uterotonics and controlled cord traction [3,14]). Apart from clinical factors, racial-ethnicity disparities and low socioeconomic status were also risk factors for PPH [15,16].

Interestingly, previous studies have reported a relatively high maternal mortality among the minorities in Thailand [17,18]. Therefore, consideration of local or context-specific risk factors, such as race, ethnicity, socioeconomic status, cultural belief, and accessibility to health care services, might be crucial [18]. In a recent systematic review, the Asian population was concluded as a definite risk factor for PPH [19]. However, there was only one Asian study in this systematic review, a study from Hong Kong by Lao et al. [20]. The study only focused on the association between maternal age and PPH and did not examine the effect of race on PPH. The other 26 studies included in the systematic review were mainly from the USA and the European countries. Therefore, the Asian population identified as risk factors was, in fact, the Asian population that was living in other non-Asian countries (e.g., USA). No studies in Thailand and other Asian countries have examined subpopulation of the Asian race as a potential risk characteristic for PPH, and most studies were conducted in tertiary care or University-affiliated hospitals located in the urban areas [5,11,19,21,22,23].

Therefore, there remains a gap of evidence regarding PPH risk factors for hospitals in the border area where ethnic diversity is usually found. This study aimed to assess the incidence and relevant risk factors for PPH among pregnant women who underwent transvaginal delivery at a Thai–Myanmar border hospital in Northern Thailand. Special attention was given to the effect of ethnicity and minority groups on the risk of PPH, as no studies have provided the answer to this particular question.

## 2. Materials and Methods

### 2.1. Design and Setting

An exploratory risk factor research was conducted with a retrospective observational nested case-control design. The primary aim of the study was to identify significant determinants of PPH in the source population of pregnant women who underwent vaginal delivery at Maesai Hospital between 2014 and 2018. Maesai hospital is an upper medium-sized community hospital with 120 in-patient beds located in the Maesai district, the northernmost district of Chiang Rai province, and is a major border crossing between Thailand and Myanmar [24]. Approximately 1000 women deliver at Maesai hospital each year. In Thailand, most community hospitals do not have obstetricians in service. Therefore, complex cases (e.g., multiple pregnancies or twin and pregnant women with multiple comorbidities) are generally referred to Chiang Rai Prachanukroh hospital, the main tertiary care center of the province.

### 2.2. Cases and Controls

**Figure 1:**
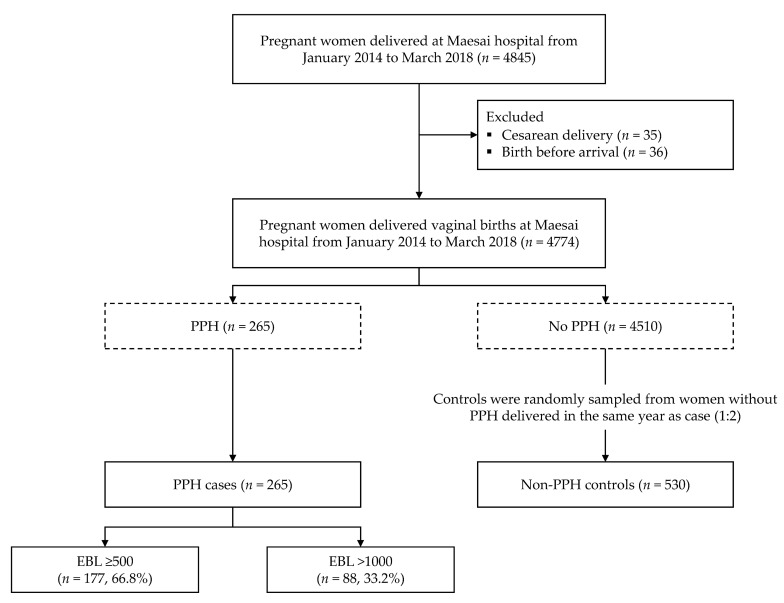
Patient flow diagram and the matching of cases and controls. Abbreviations: EBL, estimated blood loss; PPH, postpartum hemorrhage.

During the study period, we identified a cohort of 4845 women delivered at the hospital. Women who received a caesarean section or women who delivered before arrival to the hospital were excluded. In this study, we employed two definitions of PPH, which were PPH and severe PPH. PPH is defined as a blood loss of more than 500 mL within the first 24 h following delivery, while severe PPH is a blood loss of 1000 mL or more. The verification of postpartum blood loss was based on visual estimation of attending midwives from 2014 to 2015 and quantitative measurement with a collector bag from 2016 to 2018. All women with PPH during the period were defined as cases. For the selection of controls, we employed the tradition case-control approach [25], or exclusive sampling of controls [26]. All women without PPH were sampled as controls at the end of study. To ensure that all controls were selected from the same source population as cases, we first identified the cohort of women who delivered at our hospital during the study period. Hospital record numbers of all women within the cohort were collected. Cases with PPH were identified and excluded from the primary list. The remaining records in the list were used as the base for sampling of controls. For each case, two controls, or women without PPH, delivered within the same year as the PPH case were randomly sampled from the lists of non-cases. Figure 1 presents the patient flow diagram and the matching of cases and controls.

### 2.3. Data Collection

For each included patient, we reviewed routinely-documented medical and obstetric patient record forms. The data on demographic and potential risk factors were collected. These standardized forms were used for gathering clinical information from all patients upon their admissions by attending midwives. The forms record essential demographic data and potential risk characteristics for obstetrics complications encompassing all our study determinants. All the included risk factors in this study were based on a review of the evidence, clinical experience, and data available on standard record forms. Antepartum factors included maternal age, nationality, obstetrics factors (i.e., parity, antenatal care history, gestational age, maternal anemia, maternal HIV, history of previous PPH, history of previous delivery with fetal weight > 3500 gm, history of instrumental delivery, and pregnancy-induced hypertension), physical examination (i.e., BMI, fundal height, and cervical dilatation on admission). Intrapartum factors included length of each stage of labor, requirement of manual removal of placenta, type of delivery (i.e., spontaneous delivery, instrumental delivery, and Breech assisted/extraction), tear degree of the episiotomy wound, fetal weight, and estimated blood loss.

### 2.4. Definitions of Terms

Nulliparous women were women who had never gave birth (parity of zero delivery). Primiparous women were women who had gave birth once (parity of one delivery). Multiparous women were women who had gave birth more than once (parity ≥ 2 deliveries) [27]. In our study, we categorized women in terms of parity into nulliparous and non-nulliparous women, which include both primiparous and multiparous women. Maternal anemia was defined as the hemoglobin (Hb) level of less than 11 g/dL according to the World Health Organization (WHO) [28]. Prolonged second stage was defined as more than 2 h in nulliparous and more than 1 h in non-nulliparous women without epidural analgesia [29]. Prolonged third stage of labor was defined as a total length of the third stage of labor that lasts more than 30 min [30]. Requirement of manual removal of placenta might occur earlier than 30 min if there were active bleeding.

### 2.5. Statistical Analysis

All statistical analyses were performed using Stata 16 (StataCorp, College Station, TX, USA). We described categorical variables with frequency and percentage and continuous variables with mean and standard deviation, or median and interquartile range, as appropriate. Fisher’s exact probability test was used for identifying the significant difference of categorical variables between cases and controls. An independent t-test or Mann–Whitney U test was used for comparing continuous variables between cases and controls. A *p*-value of less than 0.05 was considered statistically significant.

The calculation of the overall PPH incidence was based on the number of PPH cases divided by the total number of women delivered at Maesai hospital during the entire study period. The 95% confidence interval (CI) of the overall PPH incidence was estimated with the Clopper–Pearson method.

Multivariable conditional logistic regression was used for exploratory modeling. The dependent variable was the occurrence of PPH. The independent variables were risk factors with a statistically significant difference from the univariable analysis. In our study, the verification of endpoint was different in the two periods, visual estimation from 2014 to 2015 and quantitative measurement with collector bag from 2016 to 2018, which could potentially affect the association between study determinants and the study endpoints. For this reason, we employed the concept of stratified analysis of binary logistic regression with the use of conditional logistic modeling [31]. In this study, our patients were stratified into two study periods based on the method used for measuring estimated blood loss. The study period was used as a conditional factor in a conditional logistic model. With this approach, all patients within the primary dataset were separated into two datasets by study periods and binary logistic regression were used for separate modeling. The estimates from the stratified logistic models were then combined and presented. For each PPH definition, two conditional logistic regression models were executed separately for antepartum characteristics and intrapartum characteristics. Odds ratios (OR) with their corresponding 95% CIs were presented. As the aim was to explore for independent risk factors not to explain the causality of each factor on the endpoints, all factors within the multivariable were presented regardless of their statistical significance. No model reduction or stepwise procedure was performed. To confirm the robustness of our results, we performed a sensitivity analysis using multivariable multi-level logistic regression by using the time variable as a 2nd level variable.

## 3. Results

We identified 265 women with PPH cases and 530 women with non-PPH controls during the study period. The overall incidence of PPH and severe PPH was 5.55% (95%CI 4.92% to 6.24%) and 1.84% (95% CI 1.48% to 2.27%), respectively. Supplementary Table S1 presents the PPH incidence of the patient cohort in each year. The estimated blood loss was significantly different between PPH cases and non-PPH controls (968.4 ± 431.2 (range 500–3410) vs. 257.3 ± 120.7 (range 30–495), *p*-value < 0.001). We stratified the study samples into two study periods based on the method of outcome verification. The average overall estimated blood loss between the two study periods was not significantly different (EBL 499.45 ± 452.76 vs. 485.57 ± 385.75, *p*-value = 0.660). The estimated blood loss in the PPH group was also not significantly differed between the two study periods (EBL 989.55 ± 473.40 vs. 932.30 ± 347.16, *p*-value = 0.298).

Although the mean age was not significantly different (26.3 ± 6.3 vs. 26.1 ± 5.8, *p*-value = 0.644), there was a higher proportion of teenage pregnancy (10.6% vs. 8.3%, *p*-value = 0.040) and elderly pregnancy (12.1% vs. 7.4%, *p*-value = 0.040) in women with PPH than that in women without PPH. Several antepartum factors were found to be significantly different between cases and controls in the univariable analysis, which were nationality, nulliparity, antenatal care history, history of previous PPH, history of delivering of fetal weight > 3500 gm, history of instrumental delivery, body mass index at delivery, and fundal height (Table 1). The intrapartum factors are presented in Table 2. By using univariable analysis, most factors were found to be significantly different between cases and controls. These intrapartum factors included labor augmentation, type of delivery, tear degree of the episiotomy wound, fetal weight, and estimated blood loss. Only the length of 1st stage, 2nd stage of labor, and prolongation of 2nd stage of labor were not significantly different (Table 2). There were only three cases with Breech assisted/extraction, one within PPH cases and two within non-PPH controls. Thus, we did not perform statistical comparison for this variable. Supplementary Tables S2 and S3 contrast clinical characteristics, antepartum, and postpartum factors between PPH cases and non-PPH controls based on EBL ≥ 1000 mL definition.

Five antepartum factors, including elderly pregnancy, minority groups, nulliparous, history of previous PPH, and BMI ≥ 35 kg/m^2^, were identified as independent predictors for PPH and severe PPH in multivariable analysis (Table 3). Inadequate ANC, and fundal height ≥ 36 cm were predictors for only PPH, while Burmese nationality was a predictor for only severe PPH (Table 3). Although prolonged third stage of labor and requiring manual removal of placenta were both identified as statistical significance factors in the univariable analysis, only requiring manual removal of placenta was included in the multivariable model owing to clinical collinearity. Three intrapartum factors were identified as independent predictors for PPH and severe PPH, which were requiring manual removal of placenta, labor augmentation, and fetal weight > 4000 gm (Table 4). Instrumental delivery, Third- or Fourth-degree tear of an episiotomy wound, and fetal weight between 3500 to 4000 gm were predictors for only PPH (Table 4). The sensitivity analysis results showed no significant differences in the association between predictors and PPH occurrence when considered the correlation of women delivered within the same year (Supplementary Tables S4 and S5).

## 4. Discussion

In this retrospective case-control study of women undergoing vaginal delivery at a Thai–Myanmar border hospital in Northern Thailand, the overall incidence of PPH and severe PPH was 5.55% and 1.84%, respectively. The robust risk factors for PPH and severe PPH were elderly pregnancy, minority groups, nulliparous, history of previous PPH, BMI ≥ 35 kg/m^2^, requirement of manual removal of placenta, labor augmentation, and fetal weight > 4000 gm.

In this study, the PPH incidence was higher than that of the South-east Asian countries at 4.88% [4] and much higher than other reports from Thailand, ranging from 1.98 to 2.40% [11,22]. This was in concordance with a previous report that identified a higher PPH rate in district hospitals compared to urban or tertiary care centers [5]. The higher incidence of PPH in our study could be explained by the type of clinical settings, which took place in rural areas where standard equipment and supplies are often lacked [32]. Previous studies have found that hospital levels significantly affect resource allocation and the PPH management quality [8,33].

Several antepartum risk factors in pregnant women delivered vaginally were identified in our study, which were elderly pregnancy [8,34], nulliparous [35,36], history of previous PPH [5,9,12], BMI ≥ 35 kg/m^2^ [35,37], and minority group. All these factors were supported by previous studies and were independent risk factors for both PPH and severe PPH according to our analysis. Although some factors were significant in only one of the PPH definitions, including Burmese nationality, inadequate ANC, history of instrumental delivery, and fundal height ≥ 36 cm, they should not be overlooked during an initial risk assessment of the pregnant women.

Our study population comes from a rural area on the Thai–Myanmar border with large numbers of migrants and minorities, mostly hill-tribes, which were a significant risk factor for both PPH and severe PPH in our study. This association could be multifactorial as the minorities have a specific context of socioeconomic aspect including poor economic condition, healthcare coverage and accessibility, lack of formal education, and language barrier [38]. Previous studies showed that pregnant women who live in a disadvantaged area or have low-income were at higher risk of PPH [16] and other poor maternal health outcomes [39]. Lower family income was also reported to be significantly related to unhealthy behaviors, increased maternal infection, and unintended pregnancy, leading to a lower antenatal care commitment [40].

Racial and ethnic disparities have a significant effect on both maternal morbidity, mortality, and postpartum hemorrhage [12,15,41,42]. South-East Asian ethnicity was also reported as a risk factor for PPH [43]. After adjusting for known risk factors that may bias the estimate of the effect of race, especially maternal age and BMI [44], we found that Burmese nationality was not a significant risk factor for PPH but severe PPH, which indicates that Burmese women are hemorrhaging less frequently than local Thai women, but hemorrhage appears to be more serious when they do. Biological differences may influence the effect of race or ethnicity disparities on PPH. A previous study attempting to identify genetic background that influences the PPH reported that the gene polymorphism carries a protective effect against PPH [45]. Because PPH incidence depends on various factors; genetic predisposition such as a defect in coagulation or tissue elasticity might also lead to higher PPH incidence in different races or ethnicities [46]. However, current evidence regarding genetic and racial entities as possible independent risk factors for PPH is still limited. Besides, healthcare provider bias and implicit bias may affect different levels of care to women of different racial/ethnic groups and affect doctor-patient interaction [47]. Furthermore, antenatal care visits of fewer than five times, and previous instrumental delivery history were identified as PPH risk factors. Inadequate ANC results in a lack of crucial maternal education that involves self-management and self-monitoring skills that are important for pregnant women to detect early warning signs and the physicians to identify other relevant risk factors and medical conditions that might affect the postpartum outcomes [48,49]. One study found that history of previous instrumental delivery was associated with uterine atony and increasing instrumental delivery in the following pregnancy [50].

During the intrapartum period, three factors were risk factors for PPH and severe PPH: requiring manual removal of placenta, Labor augmentation, and fetal weight more than 4000 gm, or fetal macrosomia. Not surprisingly, the strongest risk factor in our study is requiring manual removal of placenta due to retained placental tissue, which is universally known as the second-leading cause of PPH [34,51]. Augmentation of labor was also supported by many previous observational studies [52,53], as well as being consistent with one randomized control trial, which found that labor augmentation increases the volume of postpartum blood loss [54]. The hypothesized mechanism was the reduction in the uterine contractility after birth due to desensitized oxytocin receptors at the uterus [55]. Fetal macrosomia may increase postpartum blood loss via multiple pathways. The most direct mechanism is the distension of uterus due to large fetal size, which causes uterine atony after birth [56]. Studies also showed that fetal macrosomia increased the risk of instrumental delivery and third-degree tear of an episiotomy wound [8,57,58]. Other factors were significant predictors for only PPH: instrumental delivery, third- or fourth-degree tear of an episiotomy wound, fetal weight between 3500 and 4000 gm, and fundal height more than 36 cm.

The main strengths of the study are the specific domains of the patients. We collect data from Thai–Myanmar border hospitals in the Maesai district. A unique health care setting where racial and ethnicity disparities and low socioeconomic status were present and evidence was still lacking, allowing us to evaluate local or context-specific risk factors of PPH. To identify independent risk factors for PPH, we employed two definitions of PPH, both at 500 and 1000 mL. Only factors with a significant association with both PPH definitions were concluded as robust risk factors for PPH.

This study had some limitations. First, the design was case-control, and the data were retrospectively collected, which might be subjected to several types of bias (i.e., selection bias). However, as controls came from the same source population as cases and were randomly sampled from the same year, the selection bias and bias due to time effect were therefore minimized. Moreover, all the data were collected from routine, standardized records that possess all the data on essential variables. Thus, the quality of the data was adequate. In our study, observer bias was properly handled by the fact that the history of exposure to any risk factor or the occurrence of any risk that predisposes the patients to postpartum hemorrhage during the intrapartum period were recorded in a standardized routine record form by the attending midwives prior to the time of endpoint verification. Thus, the recorders of all the data on the study determinants were, at that time, blinded to the outcomes. Using of standardized risk evaluation form to gather information upon admission also eliminates the presence of recall bias, as the final status of the patient was unknown at the time of data collection. Second, the sample size may not provide adequate statistical power to identify the statistical significance of some predictors on severe PPH. However, these factors showed the same direction of the effect for PPH and were consistent with previous studies. Third, different methods to estimate postpartum blood loss were used. Visual estimation was used before 2016, whereas a standard collecting bag was used after 2016. This difference might result in differential verification bias of clinical endpoints. Regarding this issue, conditional logistic regression was used to provide the combined estimates after the stratification of patients according to their study periods. In addition, sensitivity analysis with multi-level logistic models showed consistent results to conditional logistic models. Finally, our study was conducted in a specific healthcare setting, limiting the generalizability of the results to the general population.

## 5. Conclusions

Several clinical factors were identified as independent risk factors for PPH and severe PPH in a Thai–Myanmar border community hospital, which were elderly pregnancy, nulliparous, previous PPH history, BMI ≥ 35 kg/m^2^, requiring manual removal of placenta, labor augmentation, and fetal weight > 4000 gm. Although most clinical factors identified in our study were already supported by previous evidence, our study provides additional data on the effect of race and ethnicity on the occurrence of PPH in the Asian population. Apart from other known factors, particular attention should be given to pregnant women who were minority groups as PPH risk significantly increased in this population.

## Figures and Tables

**Table 1:** Comparison of antepartum characteristics between cases and controls (n = 795).

	PPH Cases (n = 265)		No PPH Controls (n = 530)		*p*-Value
	n	(%)	n	(%)	
**Demographic factors**					
Maternal age, (years, mean ± SD)	26.3	±6.3	26.1	±5.8	0.644
Normal age pregnancy (20–34)	205	(77.4)	447	(84.3)	0.040
Teenage pregnancy (<20)	28	(10.6)	44	(8.3)	
Elderly pregnancy (≥35)	32	(12.1)	39	(7.4)	
Nationality					
Thai	45	(17.0)	138	(26.0)	<0.001
Burmese	115	(43.4)	295	(55.7)	
Minority/tribes	105	(39.6)	97	(18.3)	
**Obstetrics factors**					
Parity					
Non-nulliparous (primiparous and multiparous)	135	(50.9)	363	(68.5)	<0.001
Nulliparous	130	(49.1)	167	(31.5)	
Antenatal care history					
Have ANC history	256	(96.6)	512	(96.6)	1.000
No ANC history	9	(3.4)	18	(3.4)	
Adequacy of ANC					
Adequate (≥5 visits)	191	(72.1)	433	(81.7)	0.002
Inadequate (<5 visits)	74	(27.9)	97	(18.3)	
Gestational age					
Preterm (<37)	14	(5.3)	42	(7.9)	0.311
Term (37–41)	251	(94.7)	486	(91.9)	
Post term (>41)	0	(0)	1	(0.2)	
**Presence of the following maternal risk factors**					
Maternal anemia	45	(17.0)	80	(15.1)	0.535
Maternal HIV	0	(0)	6	(1.1)	0.186
History of previous PPH	11	(4.2)	1	(0.2)	<0.001
History of deliver fetal weight >3500 gm	25	(9.4)	25	(4.7)	0.013
History of instrumental delivery	16	(6.0)	9	(1.7)	0.002
Pregnancy-induced hypertension	7	(2.6)	7	(1.3)	0.251
**Physical examination**					
BMI at delivery, (kg/m^2^, mean ± SD)					
<25.0	70	(26.4)	157	(29.6)	<0.001
25.0–29.9	108	(40.8)	267	(50.4)	
30.0–34.9	59	(22.3)	86	(16.2)	
≥35.0	28	(10.6)	20	(3.8)	
Fundal height, (cm, mean ± SD)	35.2	±2.6	34.5	±2.3	<0.001
<36.0	168	(63.4)	447	(84.3)	<0.001
≥36.0	97	(36.6)	83	(15.7)	
Cervical dilation on admission, (cm)					
≤3 cm	173	(65.3)	340	(64.2)	0.056
4–7 cm	78	(29.4)	137	(25.9)	
≥8 cm	14	(5.3)	53	(10.0)	

Abbreviations: ANC, antenatal care; BMI, body mass index; HIV, Human immunodeficiency syndrome; PPH, postpartum hemorrhage; SD, standard deviation.

**Table 2:** Comparison of intrapartum characteristics between cases and controls (n = 795).

	PPH Cases (n = 265)		No PPH Controls (n = 530)		*p*-Value
	n	(%)	n	(%)	
**Intrapartum factors**					
Labor augmentation	72	(27.2)	64	(12.1)	<0.001
Length of 1st stage (hour, median (IQR))	8	(4.5, 13)	7	(4, 11.4)	0.085
Length of 2nd stage in nulliparous (min, median (IQR))	10	(5.5, 20)	12	(4, 21)	0.878
Length of 2nd stage in non-nulliparous (min, median (IQR))	13	(6, 25)	10	(5, 20)	0.190
Prolonged 2nd stage	9	(3.4)	23	(4.3)	0.572
Length of 3rd stage (min, median (IQR))	7	(5, 18)	6	(4, 9)	<0.001
Prolonged 3rd stage	28	(10.6)	11	(2.1)	<0.001
Require manual removal of placenta	35	(13.2)	2	(0.4)	<0.001
Delivery					
Spontaneous delivery	228	(86.0)	500	(94.3)	<0.001
Instrumental delivery	37	(14.0)	30	(5.7)	
Episiotomy wound					
No tear	200	(75.5)	420	(79.3)	<0.001
First or second degree tear	52	(19.6)	109	(20.6)	
Third or fourth degree tear	13	(4.9)	1	(0.2)	
Fetal weight (gm, mean ± SD)					
<3500	195	(73.6)	453	(85.5)	<0.001
3500–4000	62	(23.4)	73	(13.8)	
>4000	8	(3.0)	4	(0.8)	

Abbreviations: IQR, interquartile range; PPH, postpartum hemorrhage; SD, standard deviation.

**Table 3:** Multivariable analysis of association between antepartum characteristics and postpartum hemorrhage (n = 795).

	Multivariable Analysis			
	EBL ≥ 500 mL		EBL > 1000 mL	
	Adjusted OR (95% CI)	*p*-Value	Adjusted OR (95% CI)	*p*-Value
Maternal age				
Normal age pregnancy (20–34)	Reference		Reference	
Teenage pregnancy (<20)	0.94 (0.52, 1.70)	0.845	1.06 (0.46, 2.42)	0.893
Elderly pregnancy (≥35)	2.36 (1.32, 4.23)	0.004	2.82 (1.37, 5.81)	0.005
Nationality				
Thai	Reference		Reference	
Burmese	1.39 (0.88, 2.19)	0.156	2.31 (1.09, 4.89)	0.029
Minority/tribes	3.29 (2.02, 5.36)	<0.001	3.83 (1.79, 8.14)	<0.001
Parity				
Non-nulliparous (primiparous and multiparous)	Reference		Reference	
Nulliparous	3.17 (2.19, 4.59)	<0.001	2.37 (1.40, 4.00)	0.001
Adequacy of ANC				
Adequate (≥5 visits)	Reference		Reference	
Inadequate (<5 visits)	1.66 (1.11, 2.48)	0.013	1.12 (0.65, 1.92)	0.693
History of previous PPH				
Absence	Reference		Reference	
Presence	22.77 (2.82, 184.13)	0.003	11.32 (3.22, 39.77)	<0.001
History of deliver fetal weight > 3500 gm				
Absence	Reference		Reference	
Presence	1.44 (0.71, 2.89)	0.309	1.01 (0.40, 2.53)	0.976
History of instrumental delivery				
Absence	Reference		Reference	
Presence	2.39 (0.96, 5.98)	0.062	2.98 (1.14, 7.81)	0.026
BMI at delivery				
<25.0	Reference		Reference	
25.0–29.9	0.83 (0.55, 1.24)	0.361	0.93 (0.51, 1.69)	0.820
30.0–34.9	1.44 (0.89, 2.36)	0.141	1.62 (0.81, 3.16)	0.173
≥35.0	2.44 (1.15, 5.14)	0.019	3.05 (1.26, 7.37)	0.013
Fundal height				
<36.0	Reference		Reference	
≥36.0	2.97 (1.99, 4.43)	<0.001	1.69 (0.99, 2.87)	0.052

Abbreviations: ANC, antenatal care; CI, confidence interval; EBL, estimated blood loss; OR, odds ratio; PPH, postpartum hemorrhage.

**Table 4:** Multivariable analysis of association between intrapartum characteristics and postpartum hemorrhage (n = 795).

	Multivariable Analysis			
	EBL ≥ 500 mL		EBL > 1000 mL	
	Adjusted OR (95%CI)	*p*-Value	Adjusted OR (95%CI)	*p*-Value
Labor augmentation				
No	Reference		Reference	
Yes	2.34 (1.55, 3.53)	<0.001	3.50 (2.06, 5.96)	<0.001
Require manual removal of placenta				
Absence	Reference		Reference	
Presence	49.35 (11.66, 208.89)	<0.001	26.59 (12.30, 57.50)	<0.001
Delivery				
Spontaneous delivery	Reference		Reference	
Instrumental delivery	2.58 (1.48, 4.51)	0.001	1.26 (0.57, 2.76)	0.560
Episiotomy wound				
No tear	Reference		Reference	
First or second degree tear	1.16 (0.78, 1.72)	0.465	1.26 (0.70, 2.28)	0.434
Third or fourth degree tear	25.89 (3.28, 204.29)	0.002	1.24 (0.26, 5.95)	0.789
Fetal weight				
<3500	Reference		Reference	
3500–4000	2.11 (1.41, 3.16)	<0.001	1.65 (0.92, 2.98)	0.095
>4000	5.92 (1.72, 20.37)	0.005	5.42 (1.48, 19.85)	0.011

Abbreviations: CI, confidence interval; EBL, estimated blood loss; OR, odds ratio.

## Data Availability

The datasets used and/or analyzed during the current study are available from the corresponding author on reasonable request.

## Supplementary Materials

The following are available online at https://www.mdpi.com/article/10.3390/ijerph18094633/s1, Table S1: Incidence of postpartum hemorrhage of the patient cohort in each year, Table S2: Comparison of antepartum characteristics between severe PPH cases and non-severe PPH controls (n = 795), Table S3: Comparison of antepartum characteristics between severe PPH cases and non-severe PPH controls (n = 795), Table S4: Multivariable multi-level analysis of association between antepartum characteristics and severe postpartum hemorrhage (n = 795), Table S5: Multivariable multi-level analysis of association between intrapartum characteristics and severe postpartum hemorrhage (n = 795).
