# Supplementary material for: Risk Factors for Postpartum Hemorrhage in a Thai–Myanmar Border Community Hospital: A Nested Case-Control Study
Source: Int J Environ Res Public Health. 2021 Apr 27;18(9):4633. doi: 10.3390/ijerph18094633 (PMC8123817; doi:10.3390/ijerph18094633)
Supplement: Supplementary file 1 [file ijerph-18-04633-s001.zip › ijerph-1143758-supplementary.pdf]

**Supplementary Table S1.** Incidence of postpartum hemorrhage of the patient cohort

| Year | Total births | PPH (EBL ≥500 ml) | Incidence of PPH (95%CI) |
|---|---|---|---|
| 2014 | 1209 | 90 | 7.44 (6.03, 9.07) |
| 2015 | 1098 | 77 | 7.01 (5.57, 8.69) |
| 2016 | 949 | 48 | 5.06 (3.75, 6.65) |
| 2017 | 796 | 44 | 5.53 (4.04, 7.35) |
| 2018 (until March) | 722 | 6 | 0.83 (0.31, 1.80) |
| Overall | 4774 | 265 | 5.55 (4.92, 6.24) |

**Abbreviations:** CI, confidence interval; EBL, estimated blood loss; PPH, postpartum hemorrhage.

**Supplementary Table S2.** Comparison of antepartum characteristics between severe PPH cases and non-severe PPH controls (n=795).

| | PPH cases (n=88) | | No PPH controls (n=707) | | P-value |
|---|---|---|---|---|---|
| | n | (%) | n | (%) | |
| **Demographic factors** | | | | | |
| Maternal age, (years, mean±SD) | 26.3 | ±6.5 | 26.1 | ±5.9 | 0.787 |
| Normal age pregnancy (20-34) | 67 | (76.1) | 585 | (82.3) | 0.194 |
| Teenage pregnancy (<20) | 9 | (10.2) | 63 | (8.9) | |
| Elderly pregnancy (≥35) | 12 | (13.6) | 59 | (8.4) | |
| Nationality | | | | | |
| Thai | 11 | (12.5) | 166 | (23.5) | 0.001 |
| Burmese | 41 | (46.6) | 369 | (52.2) | |
| Minority/tribes | 36 | (40.9) | 166 | (23.5) | |
| **Obstetrics factors** | | | | | |
| Parity | | | | | |
| Non-nulliparous (primiparous and multiparous) | 47 | (53.4) | 451 | (63.8) | 0.062 |
| Nulliparous | 41 | (46.6) | 256 | (36.2) | |
| Antenatal care history | | | | | |
| Have ANC history | 87 | (98.9) | 681 | (96.3) | 0.348 |
| No ANC history | 1 | (1.1) | 26 | (3.7) | |
| Adequacy of ANC | | | | | |
| Adequate (≥5 visits) | 65 | (73.9) | 559 | (79.1) | 0.272 |
| Inadequate (<5 visits) | 23 | (26.1) | 148 | (20.9) | |
| Gestational age | | | | | |
| Preterm (<37) | 3 | (3.4) | 53 | (7.5) | 0.279 |
| Term (37-41) | 85 | (96.6) | 652 | (92.4) | |
| Post term (>41) | 0 | (0) | 1 | (0.1) | |
| **Presence of the following maternal risk factors** | | | | | |
| Maternal anemia | 15 | (17.1) | 110 | (15.6) | 0.756 |
| Maternal HIV | 0 | (0) | 6 | (0.9) | 1.000 |
| History of previous PPH | 7 | (8.0) | 5 | (0.7) | <0.001 |
| History of deliver fetal weight >3500 gm | 8 | (9.1) | 42 | (5.9) | 0.245 |
| History of instrumental delivery | 7 | (8.0) | 18 | (2.6) | 0.015 |
| Pregnancy-induced hypertension | 5 | (5.7) | 9 | (1.3) | 0.013 |
| **Physical examination** | | | | | |
| BMI at delivery, (kg/m$^2$, mean±SD) | | | | | |
| <25.0 | 20 | (22.7) | 207 | (29.3) | 0.004 |
| 25.0-29.9 | 34 | (38.6) | 341 | (48.2) | |
| 30.0-34.9 | 22 | (25.0) | 123 | (17.4) | |
| ≥35.0 | 12 | (13.6) | 36 | (5.1) | |
| Fundal height, (cm, mean±SD) | 35.1 | ±2.7 | 34.7 | ±2.4 | 0.210 |
| <36.0 | 58 | (65.9) | 557 | (78.8) | 0.010 |
| ≥36.0 | 30 | (34.1) | 150 | (21.2) | |
| Cervical dilation on admission, (cm) | | | | | |
| ≤3 cm | 63 | (71.6) | 450 | (63.7) | 0.366 |
| 4-7 cm | 20 | (22.7) | 195 | (27.6) | |
| ≥8 cm | 5 | (5.7) | 62 | (8.8) | |

**Abbreviations:** ANC, antenatal care; BMI, body mass index; HIV, Human immunodeficiency syndrome; PPH, postpartum hemorrhage; SD, standard deviation

**Supplementary Table S3.** Comparison of antepartum characteristics between severe PPH cases and non-severe PPH controls (n=795).

| | PPH cases (n=88) | | No PPH controls (n=707) | | P-value |
|---|---|---|---|---|---|
| | n | (%) | n | (%) | |
| **Intrapartum factors** | | | | | |
| Labor augmentation | 32 | (36.4) | 104 | (14.7) | <0.001 |
| Length of 1st stage (hour, median (IQR)) | 7 | (4.5, 13) | 7.5 | (4, 12) | 0.704 |
| Length of 2nd stage in nulliparous (min, median (IQR)) | 8 | (6, 15) | 11 | (5, 23) | 0.568 |
| Length of 2nd stage in non-nulliparous (min, median (IQR)) | 13 | (6, 25) | 10.5 | (5, 20) | 0.455 |
| Prolonged 2nd stage | 3 | (3.23) | 29 | (4.13) | 1.000 |
| Length of 3rd stage (min, median (IQR)) | 9.5 | (6, 24) | 6 | (4, 10) | <0.001 |
| Prolonged 3rd stage | 14 | (15.9) | 25 | (3.5) | <0.001 |
| Retained placenta | 25 | (28.4) | 12 | (1.7) | <0.001 |
| Delivery | | | | | |
| Spontaneous delivery | 77 | (91.6) | 651 | (92.1) | 0.154 |
| Instrumental delivery | 11 | (12.5) | 56 | (7.9) | |
| Episiotomy wound | | | | | |
| No tear | 68 | (77.3) | 552 | (78.1) | 0.825 |
| First or second degree tear | 18 | (20.5) | 143 | (20.2) | |
| Third or fourth degree tear | 2 | (2.3) | 12 | (1.7) | |
| Fetal weight (gm, mean±SD) | | | | | |
| <3500 | 66 | (75.0) | 582 | (82.3) | 0.032 |
| 3500-4000 | 18 | (20.5) | 117 | (16.6) | |
| >4000 | 4 | (4.6) | 8 | (1.1) | |

**Abbreviations:** IQR, interquartile range; PPH, postpartum hemorrhage; SD, standard deviation.

**Supplementary Table S4.** Multivariable multi-level analysis of association between antepartum characteristics and severe postpartum hemorrhage (n=795)

| | Multivariable analysis | | | |
|---|---|---|---|---|
| | EBL ≥ 500 ml | | EBL > 1000 ml | |
| | Adjusted OR (95%CI) | P-value | Adjusted OR (95%CI) | P-value |
| Maternal age | | | | |
| Normal age pregnancy (20-34) | Reference | | Reference | |
| Teenage pregnancy (<20) | 0.93 (0.52, 1.68) | 0.813 | 1.08 (0.47, 2.47) | 0.855 |
| Elderly pregnancy (⩾35) | 2.36 (1.32, 4.23) | 0.004 | 2.02 (0.93, 4.35) | 0.074 |
| Nationality | | | | |
| Thai | Reference | | Reference | |
| Burmese | 1.37 (0.87, 2.16) | 0.169 | 2.18 (1.03, 4.63) | 0.042 |
| Minority/tribes | 3.25 (2.00, 5.28) | <0.001 | 3.30 (1.55, 7.06) | 0.002 |
| Parity | | | | |
| Non-nulliparous (primiparous and multiparous) | Reference | | Reference | |
| Nulliparous | 3.18 (2.20, 4.62) | <0.001 | 2.26 (1.33, 3.84) | 0.003 |
| Adequacy of ANC | | | | |
| Adequate (≥5 visits) | Reference | | Reference | |
| Inadequate (<5 visits) | 1.68 (1.13, 2.51) | 0.011 | 1.06 (0.61, 1.86) | 0.831 |
| History of previous PPH | | | | |
| Absence | Reference | | Reference | |
| Presence | 23.07 (2.84, 187.11) | 0.003 | 12.58 (3.60, 43.92) | <0.001 |
| History of deliver fetal weight >3500 gm | | | | |
| Absence | Reference | | Reference | |
| Presence | 1.44 (0.71, 2.89) | 0.309 | 1.01 (0.40, 2.53) | 0.976 |
| History of instrumental delivery | | | | |
| Absence | Reference | | Reference | |
| Presence | 1.42 (0.71, 2.86) | 0.321 | 0.97 (0.37, 2.51) | 0.948 |
| BMI at delivery | | | | |
| <25.0 | Reference | | Reference | |
| 25.0-29.9 | 0.83 (0.55, 1.24) | 0.360 | 1.01 (0.55, 1.86) | 0.975 |
| 30.0-34.9 | 1.45 (0.89, 2.37) | 0.135 | 1.84 (0.92, 3.69) | 0.083 |
| ⩾35.0 | 2.45 (1.16, 5.16) | 0.019 | 3.08 (1.25, 7.60) | 0.015 |
| Fundal height | | | | |
| <36.0 | Reference | | Reference | |
| ⩾36.0 | 2.97 (1.99, 4.44) | <0.001 | 1.66 (0.97, 2.85) | 0.064 |

**Abbreviations:** ANC, antenatal care; CI, confidence interval; EBL, estimated blood loss; OR, odds ratio; PPH, postpartum hemorrhage.

**Supplementary Table S5.** Multivariable multi-level analysis of association between intrapartum characteristics and severe postpartum hemorrhage (n=795)

| | Multivariable analysis | | | |
|---|---|---|---|---|
| | EBL ≥ 500 ml | | EBL > 1000 ml | |
| | **Adjusted OR (95%CI)** | **P-value** | **Adjusted OR (95%CI)** | **P-value** |
| Labor augmentation | | | | |
| No | Reference | | Reference | |
| Yes | 2.30 (1.53, 3.47) | <0.001 | 3.55 (2.03, 6.23) | <0.001 |
| Retained placenta | | | | |
| Absence | Reference | | Reference | |
| Presence | 50.15 (11.83, 212.45) | <0.001 | 28.94 (13.24, 63.24) | <0.001 |
| Delivery | | | | |
| Spontaneous delivery | Reference | | Reference | |
| Instrumental delivery | 2.53 (1.46, 4.40) | 0.001 | 1.40 (0.64, 3.08) | 0.404 |
| Episiotomy wound | | | | |
| No tear | Reference | | Reference | |
| First or second degree tear | 1.15 (0.77, 1.71) | 0.490 | 1.28 (0.70, 2.36) | 0.427 |
| Third or fourth degree tear | 26.35 (3.34, 207.67) | 0.002 | 1.32 (0.27, 6.42) | 0.733 |
| Fetal weight | | | | |
| <3500 | Reference | | Reference | |
| 3500-4000 | 2.13 (1.42, 3.20) | <0.001 | 1.55 (0.84, 2.87) | 0.162 |
| >4000 | 5.87 (1.70, 20.26) | 0.005 | 5.97 (1.62, 21.98) | 0.007 |

**Abbreviations:** CI, confidence interval; EBL, estimated blood loss; OR, odds ratio.
